# Propensity Score Modeling in Electronic Health Records with Time-to-Event Endpoints: Application to Kidney Transplantation

**DOI:** 10.6339/22-jds1046

**Published:** 2022-04-20

**Authors:** Jonathan W. Yu, Dipankar Bandyopadhyay, Shu Yang, Le Kang, Gaurav Gupta

**Affiliations:** 1 Department of Biostatistics, Virginia Commonwealth University, Richmond, VA, USA; 2 Department of Statistics, North Carolina State University, Raleigh, NC, USA; 3 Division of Nephrology, Virginia Commonwealth University, Richmond, VA, USA

**Keywords:** cure rate, frailty, heavy censoring, inverse probability weighting, kidney transplantation, propensity scores

## Abstract

For large observational studies lacking a control group (unlike randomized controlled trials, RCT), propensity scores (PS) are often the method of choice to account for pre-treatment confounding in baseline characteristics, and thereby avoid substantial bias in treatment estimation. A vast majority of PS techniques focus on average treatment effect estimation, without any clear consensus on how to account for confounders, especially in a multiple treatment setting. Furthermore, for *time-to event* outcomes, the analytical framework is further complicated in presence of high censoring rates (sometimes, due to non-susceptibility of study units to a disease), imbalance between treatment groups, and clustered nature of the data (where, survival outcomes appear in groups). Motivated by a right-censored kidney transplantation dataset derived from the United Network of Organ Sharing (UNOS), we investigate and compare two recent promising PS procedures, (a) the generalized boosted model (GBM), and (b) the covariate-balancing propensity score (CBPS), in an attempt to decouple the causal effects of treatments (here, study subgroups, such as hepatitis C virus (HCV) positive/negative donors, and positive/negative recipients) on time to death of kidney recipients due to kidney failure, post transplantation. For estimation, we employ a 2-step procedure which addresses various complexities observed in the UNOS database within a unified paradigm. First, to adjust for the large number of confounders on the multiple sub-groups, we fit multinomial PS models via procedures (a) and (b). In the next stage, the estimated PS is incorporated into the likelihood of a semi-parametric cure rate Cox proportional hazard frailty model via inverse probability of treatment weighting, adjusted for multi-center clustering and excess censoring, Our data analysis reveals a more informative and superior performance of the full model in terms of treatment effect estimation, over sub-models that relaxes the various features of the event time dataset.

## 1 Introduction

In observational studies, the processes determining individual treatments (or exposures) may also directly impact the outcomes of interest. There, the data covariables may confound treatment selection, which hinders estimation of the true causal estimates of the exposures, leading to selection bias (Rosenbaum and Rubin, 1983). In other words, a subject’s probability of receiving a treatment in an observational/non-randomized setting remains unknown, and will depend on both observed and unobserved covariates. This is in contrast to randomized control trials (RCTs), where the goal of randomization is to balance treatment groups (i.e., all subjects have the equal probability of being assigned to a particular treatment condition) on any confounding factors (whether observed or unobserved), eliminating treatment selection bias, and ensuring that the groups are comparable (Morgan, 2018). To mitigate this, propensity score (PS) methods (Rosenbaum, 2010) have evolved, which estimates the conditional probability of treatment assignments adjusting for various (observed) baseline pre-exposure covariates. Thus, confounder balancing becomes precedent in PS analysis (Li et al., 2018), enabling comparisons of outcomes between treatment groups with similar distributions of observed covariates.

Traditional PS methods of controlling for similar distributions and estimating the average causal effect E(Δ), where $\Delta =Y_{1}-Y_{0}$, with $Y_{1}$ and $Y_{0}$ representing outcome measures for a subject enrolled in the treatment and control groups, respectively, include stratification (Neuhäuser et al., 2018), regression adjustment (Lunceford and Davidian, 2004), matching (Stuart, 2010), and weighting (Hirano and Imbens, 2001). When multiple confounding variables are involved, simple stratification methods are not ideal, given that the number of strata to adjust for increases while the samples per stratum would become sparse. Additionally, turning continuous variables into discrete cases is not ideal. Also, covariate-adjusted regression methods depend on the correct model selection, and underlying model assumptions. Of the remaining two methods, there is a rich literature with respect to weighting (Imbens, 2000; Hirano and Imbens, 2001; Crump et al., 2006; Li et al., 2018) and matching (Austin, 2009; Rosenbaum, 2010; Ma and Wang, 2020). The weighting case borrows ideas from sample surveys, where each sample unit of the treatment groups are inversely weighted by the estimated PS (popularly called, the inverse probability treatment weighted, or IPTW estimation), such that comparisons can be made between the weighted outcomes. Our current focus is exploring the *weighting* method, now for clustered/multi-level, right-censored survival outcomes.

The context for this work comes from a kidney (organ) transplantation dataset derived from the United Network of Organ Sharing, UNOS (Dharnidharka et al., 2005). With a relatively low supply of healthy (disease-free) organs readily available to match the increasing demand, the transplantation community has been pressured to search for organs from other sources. For example, hepatitis C virus (HCV) has long been recognized as a major cause for kidney diseases (Barsoum et al., 2017), and traditionally, HCV positive donor (D+) kidneys were not offered to HCV negative recipients (R−) on the waiting list, in at attempt to avoid viral transmission (Pereira et al., 1995; Bouthot et al., 1997). However, motivated by positive results (Testa and Siegler, 2014) in recent times, transplant centers have been expanding their acceptance pool to include offering HCV+ donor organs to HCV+ recipients (R+), thereby decreasing the waiting time for compatible kidneys (Kucirka et al., 2010; Bucci et al., 2004). It has been shown that the D+/R+ pairing have improved survival on the overall, without significant worsening for some transplants (Maluf et al., 2007). Similarly, it was also shown that (D+) kidneys could be offered to HCV− recipients (R−), i.e., similar survival experience was observed (Gupta et al., 2017), compared to matched counterparts of D−/R− pairings, despite the likelihood of viral transmission.

**Figure 1:**
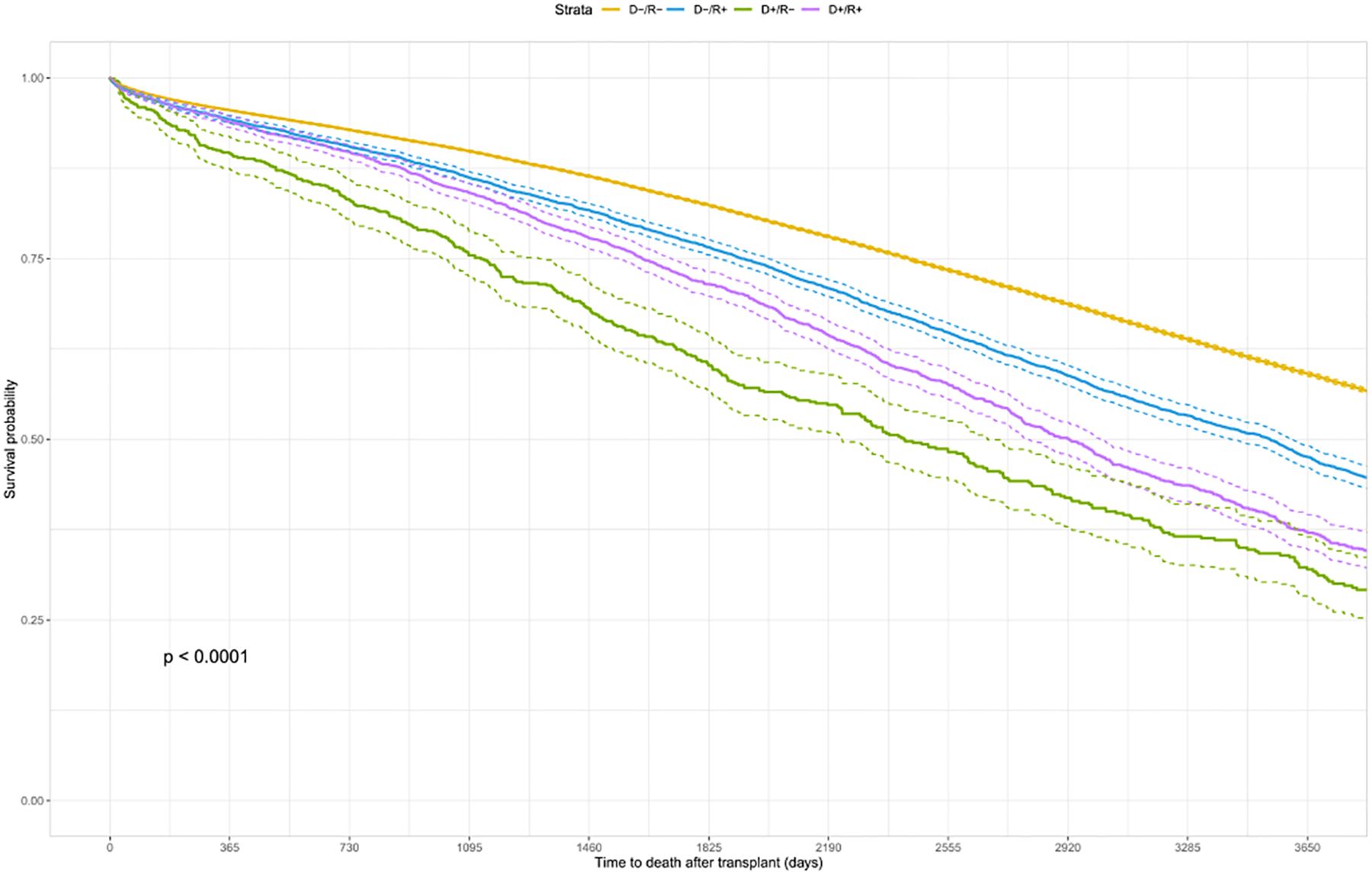
Kaplan-Meier curves depicting 10-year survival performance of transplant recipients, corresponding to the HCV± donor and recipient pairings. Solid lines denote the survival curves while dotted lines denote the 95% confidence intervals. HCV negative donors (D−) with HCV negative recipients (R−) pairing exhibited the highest survival, whereas the HCV positive donor (D+) with HCV negative recipient (R−) pairing had the lowest survival.

In this paper, we attempt to unravel the causal (exposure) effects of the various HCV± donor/recipient pairings (HCVDR), compared to the D−/R− (baseline) group, on the (increased) risk in the transplant recipient’s death in the UNOS dataset. However, there exists additional challenges (Mao et al., 2018) in implementing the PS weighting method for survival outcomes under heavy-censoring patterns, clustered nature of the data, and exposure group imbalance. Apparently, due to the right-censored nature of the outcomes, typical causal quantities, such as average causal effect (mean difference between survival times between exposure groups) may not be estimable, leading to challenges in the definition of the estimand. Also, the IPTW estimates can be unstable (Crump et al., 2009), when there is lack of overlap in the PS distribution between the exposure groups. In addition, the heavy-censoring observed can mostly be attributed to the existence of a ‘cured’ proportion – the long-term survivors who are non-susceptible to the event of interest, i.e., patient failure. For example, the estimated 10-year Kaplan-Meier survival plots indicate heavy censoring in the tails (see, Figure 1), with an overall censoring rate of 68.4%. Applying the simple nonparametric Maller and Zhou test (Maller and Zhou, 1992), the p-value of 1.42 × 10^−21^ provide significant evidence of the existence of a cure fraction. Furthermore, the donor/recipient combinations are nested within transplant centers across the US, leading to a clustered/multi-level framework, whereas, a majority of PS techniques have been developed under independence assumptions among the outcomes (Rosenbaum and Rubin, 1983). As such, a proper statistical framework is desired to address these issues, and derive appropriate interpretation for survival outcomes. To mitigate these, we propose a unified two-step approach. The first step estimates PS for the exposure groups, where we compare two recent promising approaches – the generalized boosted model, GBM (McCaffrey et al., 2013), and the covariate balancing PS, CBPS (Imai and Ratkovic, 2014) method. In the second step, we initiate the IPTW approach, where the estimated PS enter the likelihood of a Cox proportional hazard (CPH) model, adjusted for cured subjects, and a frailty term to handle the multi-level structure.

The rest of the paper proceeds as follows. We start with a short description of the motivating UNOS data in Section 2, leading to the two-step modeling approach in Section 3. While Section 4 outlines the estimation details, Section 5 illustrates our methodology via application to the UNOS data. Finally, Section 6 concludes, with some discussion.

## 2 Motivating UNOS Data

Since 1987, the Organ Procurement and Transplantation Network (OPTN), through the UNOS organization, has maintained a large national registry of all organ transplant recipients and waitlist registrants across United States (US). In addition to information on the living and deceased donors, information is recorded via one of three forms: the Transplant Candidate Registration (TCR), the Transplant Recipient Registration (TRR), and the Transplant Recipient Follow-up (TRF). The TCR includes information at time of listing such as demographic data, prior transplant data, etc. The TRR records information on the initial transplant admission, such as pre-transplant clinical data, infectious disease status, post-transplant data, etc. Finally, TRF records information at each visit following transplant, such as cause of death (if applicable), and other clinical information. The outcome of interest in this study is ‘Time to patient death, 10 years post kidney transplant’. It should be noted that patient death does not necessitate death due to kidney failure. The set of available covariates include demographic records such as donor and recipient gender, age, race (Caucasians, African Americans, or others), and a number of clinical variables, such as (hospital) length of stay (LOS), peak panel reactive antibodies (PRA), number of prior kidney transplants, blood type, hours of kidney in cold ischemia, post-transplant delayed graft function (DGF), peripheral vascular disease (PVD), diabetes, creatinine at discharge, and calculated kidney donor risk index (KDRI). Most of these covariates were adjusted or matched in prior kidney studies (Gupta et al., 2017), or adjusted for in simultaneous organ transplants studies (Gill et al., 2009; Kamal et al., 2020); however, they failed to account for the high censoring (possible high probability of survival from transplantation after a few years), and (clustered) multi-center data (i.e., many transplant centers across the US), while evaluating the survival performances of the exposure subgroups.

## 3 Two-Stage Model

### 3.1 Potential Outcomes Framework

In our setup, we consider every recipient/subject $j,j=1,\ldots ,n_{i}$ nested within the transplant center i,i=1,…,I to be undergoing one (kidney) transplant. This implies center i conducts $n_{i}$ transplants, such that $\sum_{i=1}^{I}n_{i}=N$, the total number of transplants. Let again, $W_{ij}={\left(W_{ij,1},\ldots ,W_{ij,p}\right)}^{T}$ be a p×1 vector of fixed effect confounders for subject j within center i, with the corresponding parameter vector γ. In this paper, we refrain from evaluating possible time-varying covariates to avoid complicating the setup further. We follow the potential outcomes framework (Rubin, 2005) to define the causal effects of treatments (here, the 4 groups specified by D±/R±). Let $D_{ij}(v)$ and $C_{ij}(v)$ be the potential failure and censoring time, respectively, for subject j within center i in group v, where v∈{D±/R±}. Thus, for each subject, there are four potential failure and four potential censoring times. The potential outcomes are mathematical constructs; however, they are useful to define the causal effects of treatment. We describe the causal model of the potential outcomes in Section 3.3.

Let $V_{ij}$ be the observed treatment status. Now, following a typical right-censored setup (and suppressing v), the observed data is $\left(X_{ij},\delta_{ij}\right)$, where $X_{ij}=\text{min}\left(D_{ij},C_{ij}\right),\delta_{ij}=I\left(D_{ij}⩽C_{ij}\right)$, where δ is the censoring indicator, and I(⋅), the indicator function. We now present the necessary assumptions to proceed with out setup.

**Assumption 1** (Causal consistency). $X_{ij}=X_{ij}\left(V_{ij}\right)$ *and* $C_{ij}=C_{ij}\left(V_{ij}\right)$.

**Assumption 2** (No unmeasured confounders). $\left\{D_{ij}(v),C_{ij}(v)\right\}⊥V_{ij}|W_{ij}$

**Assumption 3** (Sufficient Overlap). $0<P\left(V_{ij}=v|W_{ij}\right)<1$, *for all* v∈{D±/R±}

**Assumption 4** (Independent censoring). $D_{ij}(v)⊥C_{ij}(v)|\left(V_{ij},W_{ij}\right)$

Assumption 1 links the observed outcome to the potential outcomes. The fundamental problem in causal inference is that not all potential values can be observed, and thus the causal effects of treatments (groups) are not identifiable without further assumptions 2–4. These are made to draw valid causal conclusions with the observed data. Assumption 2 requires the covariates $W_{ij}$ to be rich enough to capture all confounders of treatment and outcome. Assumption 3 indicates that all subjects have positive probabilities of receiving any treatment/group assignment v. Assumption 4 is plausible, if $\left(V_{ij},W_{ij}\right)$ explains the censoring mechanism well. Now, under Assumptions 1–4, the causal model for the potential failure time can be identified based on the observed data; see Section 3.3. We are now in a position to present our two-stage modeling.

### 3.2 Stage I: Propensity Modeling

Using standard definitions (Rosenbaum, 2010), the PS in our time-invariant multiple exposures setup (4 groups: D± /R±) for group v is given as $e_{ij}^{\left(v\right)}=P\left(V_{ij}=v|W_{ij}\right)$, where $V_{ij}$ is the exposure assignment indicator. A common method to estimate the PS is to use a multinomial probit/logistic regression (Dow and Endersby, 2004), with the PS generated as the predicted probabilities. The inverse of the propensities would then be used as weights $\pi_{ij}^{\left(v\right)}=\frac{1}{e_{ij}^{\left(v\right)}}$ in the corresponding data likelihoods. Under strong ignorability assumptions (Rosenbaum and Rubin, 1983) for exposure assignments, we assume all confounders are captured via W, and there is a positive probability of membership among the exposure groups for all values of W, i.e., $0<e_{ij}^{\left(v\right)}<1$ for all v. Thus, the estimated PS is all that is required to control for pre-exposure differences (Stuart, 2010). Various other weighting schemes include *matching* (Li and Greene, 2013), *overlapping* (Mao et al., 2018), and *truncated/trimming* (Crump et al., 2006; Yang and Ding, 2018) for further covariate balancing, in cases of poor-overlap for dichotomous and possibly multiple groups. Next, we describe two popular methods, the GBM and CBPS, both of which were developed to optimize the PS estimation, leading to the desired weighted balance.

#### GBM:

The GBM (McCaffrey et al., 2013) is a nonparametric method that utilizes an iterative tree-based machine learning algorithm, specifically, a combination of regression trees and boosting, to estimate the probability of dichotomous, or multiple treatment assignments. For the dichotomous case, the GBM algorithm proceeds via iterative fits, starting from a globally constant model, and adds a simple regression tree at each step that provide the best fit to the residuals of the model from the previous step. A regression tree, defined as a ‘forward stagewise additive algorithm’, induces a split (or tree branch) iteratively to achieve the largest likelihood increase among all possible splits. With each addition, an increasingly complex piecewise-constant function is created. Utilizing boosting (Friedman et al., 2000), the GBM combines multiple trees, resulting in a smoother fit with better prediction than a simple regression tree alone, as well as reducing over-fitting (Burgette et al., 2016). The recursive splits within the GBM implementation automatically accommodates non-linearity and interaction effects, while the cumulative effect of multiple splits may describe complex relationships between the confounders and the exposure group assignment (Loh, 2011). It is also robust to missing data and outliers, and provides a stable estimation of the PS weighted estimators by flattening out at the extreme values, i.e., values close to 0 or 1.

Along the lines of McCaffrey et al. (2004, 2013), we now provide a brief algorithm (see Algorithm 1) for implementing GBM under multiple exposure groups. First, the algorithm creates dummy indicators $Z_{ij,v}$ corresponding to treatment/group v for subject j in center i, and where $Z_{ij,v}$ is an element of the vector $Z_{ij}={\left(Z_{ij,1},\ldots ,Z_{ij,V-1}\right)}^{\prime }$. It then models the log-odds of the exposure assignments $g^{\left(v\right)}(W)=\text{logit}\left\{e^{\left(v\right)}(W)\right\}$ using simple regression trees, where $e^{\left(v\right)}(W)$ is the propensity probability for the v-th exposure/group. The algorithm first sets the initial log-odds estimate ${\overset{ˆ}{g}}_{0}^{\left(v\right)}(W)$ to be $\text{logit}(\overset{‾}{Z})$, where $\overset{‾}{Z}$ is the average treatment assignment indicator for the whole sample. It then “boosts” the model by iteratively adding small adjustments θ to the initial estimates that would improve the model fit. The small adjustment θ uses simple regression trees that uses the residuals $r_{ij}=Z_{ij,v}-\frac{1}{1+\text{exp}\left[-\overset{ˆ}{g}\left(W_{ij}\right)\right]}$ for the current PS estimate $\frac{1}{1+\text{exp}\left[-\overset{ˆ}{g}\left(W_{ij}\right)\right]}$. This step is equivalent to adding the derivative of the log-likelihood to maximize the log-likelihood function. Separate GBMs were fitted to each exposure (group) indicators (more explanation in Section 4) to obtain estimated PS for the given exposure. The estimated PS are calculated at the iteration which achieves the best “balance” measures between each treatment and the pooled sample(s) from all treatments, using the statistic *standardized bias*, or absolute standardized mean difference (ASMD), defined as the $\frac{\left|{\overset{‾}{W}}_{ij,pv}-{\overset{‾}{W}}_{ij,pV}\right|}{{\overset{ˆ}{\sigma }}_{ij,p}}$, where ${\overset{‾}{W}}_{ij,pv}=\sum_{i=1}^{I}\sum_{j=1}^{n_{i}}\left(\frac{Z_{ij,v}W_{ij,p}}{{\overset{ˆ}{e}}_{ij}^{\left(v\right)}}\right)/\left(\frac{Z_{ij,v}}{{\overset{ˆ}{e}}_{ij}^{\left(v\right)}}\right),{\overset{‾}{W}}_{ij,pV}=\frac{1}{V}\sum_{v=1}^{V}{\overset{‾}{W}}_{ij,pv}$, the (unweighted) mean corresponding to confounder p for the pooled sample across all exposures, ${\overset{ˆ}{\sigma }}_{ij,p}$, the (unweighted) standard deviation of confounder p for the pooled sample, and ${\overset{ˆ}{e}}_{ij}^{\left(v\right)}$ the estimated PS for treatment v for subject j in center i. Another important statistic employed for balance assessment is the *Kolomogorov-Smirnov* (KS) statistic, defined as ${\text{sup}}_{w}\left|\frac{\sum_{i=1}^{I}\sum_{j=1}^{n_{i}}1\left\{W_{ij,pv}⩽w\right\}}{n}-\frac{\sum_{i=1}^{I}\sum_{j=1}^{n_{i}}1\left\{W_{ij,pv}⩽w\right\}}{n}\right|$, which is the maximum vertical distance between the unweighted empirical cumulative distribution function (EDF) for the exposure sample and the pooled sample. The PS generated via GBM has been demonstrated to outperform (Lee et al., 2010) other competing methods.

Despite its potential, major detriments of the GBM method include proper specifications of the covariates, the model complexity, and associated model misspecification, which can affect the PS estimation, and ultimately, the mean square errors (MSEs) and bias for evaluating exposure effects (Kang et al., 2007). The search for an appropriately estimated PS leads to achieving covariate balancing (Imai et al., 2008), and in that vein, the development of a robust method called covariate balancing propensity score, or CBPS (Imai and Ratkovic, 2014), which we describe next.

**Algorithm 1:** Algorithm for GBM (adapted from McCaffrey et al. (2004, 2013).

1:	First create dummy indicators for each of the V treatments, $Z_{\text{ij},v}$
2:	Set initial log-odds estimate to be ${\overset{ˆ}{g}}_{0}^{\left(v\right)}\left(W_{\text{ij}}\right)=\text{log}\{p(\overset{‾}{Z})/[1-\overset{‾}{Z}]\}$, where $\overset{‾}{Z}$ is the average treatment assignment indicator for entire sample
3:	**procedure** Regression tree-based method for each exposure indicator **for:** m = 1,…, M iterations **do:**
4:	Let $r_{\text{ij}}=Z_{\text{ij},v}-1/\left\{1+\text{exp}\left[-{\overset{ˆ}{g}}_{m-1}^{\left(v\right)}\left(W_{\text{ij}}\right)\right]\right\}$
5:	Construct a tree-structure predictor of $r_{\text{ij}}$ to partition the features into terminal nodes $T_{1},\ldots ,T_{k}$
6:	Compute the updates for each terminal node $\theta_{k}=\frac{\sum_{{\text{w}}_{\text{ij}}\in T_{k}}Z_{\text{ij},v}-e({\text{W}}_{\text{ij}})}{\sum_{{\text{w}}_{\text{ij}}\in T_{k}}p({\text{W}}_{\text{ij}})[1-e({\text{W}}_{\text{ij}})]}$
7:	Update the logistic regression model as ${\overset{ˆ}{g}}_{m}^{\left(v\right)}(W)\leftarrow {\overset{ˆ}{g}}_{m-1}^{\left(v\right)}(W)+\eta \theta_{k(W)}$ where η∈(0,1] is a shrinkage coefficient and k(W) indicates the covariate space for the k-th terminal node
8:	**end procedure**

#### CBPS:

The CBPS, an alternative to the GBM, presents robustification to the potential misspecification of a parametric PS by directly incorporating the key covariate balancing property into the estimation framework. The novelty lies in determining the conditional probability of treatment assignment and covariate balancing score in a single estimation call via a set of moment conditions. The estimation is carried out utilizing the generalized method of moments, GMM (Hansen, 1982), or the empirical likelihood (EL) framework (Owen, 1991). Following Imai and Ratkovic (2014), the estimated generalized propensity score (GPS) for V treatments can be written as multinomial probabilities $e_{\text{ij}}^{\left(v\right)}\left(W_{\text{ij}}\right)=\text{Pr}\left(V_{\text{ij}}=v|W_{\text{ij}}\right)$, such that the conditional probabilities sum to 1 (Imbens, 2000). A multinomial logistic regression can be employed to model the GPS. The corresponding moment conditions under the score function using the likelihood framework is given as

$$\frac{1}{N}\sum_{i=1}^{I}\sum_{j=1}^{n_{i}}\sum_{v=1}^{V}\left[\frac{1\left\{V_{\text{ij}}=v\right\}}{e_{\text{ij}}^{\left(v\right)}\left(W_{\text{ij}}\right)}\cdot \frac{\partial e_{\text{ij}}^{\left(v\right)}\left(W_{\text{ij}}\right)}{\partial \gamma^{T}}\right]=0$$

where, γ is the column vector of unknown parameters. As such, this condition results in V-1 set of moments:

(1) $$\frac{1}{N}\sum_{i=1}^{I}\sum_{j=1}^{n_{i}}\left[\frac{1\left\{V_{\text{ij}}=v\right\}{\tilde{W}}_{\text{ij}}}{e_{\text{ij}}^{\left(v\right)}\left(W_{\text{ij}}\right)}-\frac{1\left\{V_{\text{ij}}=v-1\right\}{\tilde{W}}_{\text{ij}}}{e_{\text{ij}}^{\left(v-1\right)}\left(W_{\text{ij}}\right)}\right]=0$$

for v=2,…,V and where ${\tilde{W}}_{\text{ij}}=f\left(W_{\text{ij}}\right)$ is a p-dimensional vector of potential confounder covariates. These moment conditions can be combined with the score function under the GMM or EL framework. Following Hansen (1982), the GMM estimator is given as

$${\overset{ˆ}{\gamma }}_{\text{GMM}}=\text{arg}\;\underset{\gamma \in \Theta }{\text{max}}\;{\overset{‾}{g}}_{\gamma }(V,W{)}^{T}\Sigma_{\gamma }(V,W{)}^{-1}{\overset{‾}{g}}_{\gamma }(V,W)$$

with the sample mean of moment conditions ${\overset{‾}{g}}_{\gamma }(V,W)=\frac{1}{N}\sum_{i=1}^{I}\sum_{j=1}^{n_{i}}g_{\gamma }\left(V_{\text{ij}},W_{\text{ij}}\right)$, where,

gγVij,Wij=1Vij=vW~ijeij(v)Wij-1Vij=v-1W~ijeij(v-1)Wij1N∑i=1I∑j=1ni1Vij=v-eij(v)WijW~ijeij(v)Wij1-eij(v)Wij

with covariance estimator $\Sigma_{\text{GMM}}(V,W)=\frac{1}{N}\sum_{i=1}^{I}\sum_{j=1}^{n_{i}}\text{E}\left\{g_{\gamma }\left(V_{\text{ij}},W_{\text{ij}}\right)g_{\gamma }{\left(V_{\text{ij}},W_{\text{ij}}\right)}^{T}|W_{\text{ij}}\right\}$, which penalizes large weights. A gradient-based optimization method is then used; for more details, see Imai and Ratkovic (2014).

In their extensive study, Setodji et al. (2017) provide guidelines in choosing between these two superior propensity estimation methods (compared to the conventional logistic regression approach), found that the GBM tends to perform better in MSEs and bias for more complex models and under proper choices of parameters. The GBM tunes the complexity by optimizing the covariate balance between the inverse probability weighted treatments cases and its lack of assumption for linearity in unknown parameters accommodate complex interactions between parameters. On the other hand, if the outcome models are linear in nature with the covariates and the general logistic regression assumptions are met, then the CBPS would perform better balance. This current study does not focus on conducting extensive comparison between the two methods, but merely to provide a framework to conduct time-to-event analysis while addressing common problems that can occur in electronic health records.

#### Common Support Assessment:

In studies involving PS methods, common support assessments are critical via evaluating the overlap of the PS distributions between exposure groups. This overlap indicates the potential for a member (study subject) within an exposure group to be in the other groups, and subjects with each covariate levels can exhibit exposure status to any groups, thereby satisfying the exchangeability and positivity assumptions (Hernán and Robins, 2006; Vaughan et al., 2015; Ma and Wang, 2020; D’Amour et al., 2021). Note, a lack of common support, or complete separation of PS between the exposure groups indicates inappropriateness of the PS methods to account for confounding. Common support is mostly assessed via visualizing the distributions (box-plots) of the PS for each exposure groups.

To assess the overlap for the GBM, box-plots of the distributions of the estimated PS for each of the transplant’s exposure group (4 groups in total) were obtained from the GBM fit, and compared. After the GBM estimates the PS model for each exposure group, D−/R−, D−/R+, D+/R−, D+/R+, PS are calculated for each of the transplants in the data, regardless of their actual assignment. The distributions of the estimated PS are presented as a separate box-plot for each transplant receiving each exposure. For example, after the GBM estimates the PS model for D−/R−, side-by-side box-plots are created from the estimated PS values for each of the four exposure groups. These are done in a similar fashion for the other three exposure groups: D−/R+, D+/R−, and D+/R+.

#### Balance Assessment:

Following common support validation, the next step is to assess the degree to which confounding by the modeled factors has been controlled for. This is the crucial balance assessment step, and is mostly achieved via calculating summary metrics, such as, the standardized bias, or KS statistics (as described earlier) for the original (unweighted) sample, and the sample with the IPTW PS adjustments, and setting a threshold. For example, a standardized bias < 0.25 would signal achieving proper balance of the potential confounder between exposure groups, while exceeding the threshold would be indicative of residual confounding (Harder et al., 2010; Vaughan et al., 2015). For the KS, the corresponding (popular) threshold is 0.1 (Stuart et al., 2013). Although the GBM fitting (corresponding to each binary exposure groups) automatically checks group balancing, we need to achieve overall balance across multiple groups. To produce an overall summary following McCaffrey et al. (2013), we consider the maximum of the balancing metrics corresponding to each exposure groups. An elegant graphical representation of this is achieved via the “Love plot”, named after Dr. Thomas E. Love (Ahmed et al., 2008), implemented in the R cobalt package (Greifer, 2020). Regarding the choice of metrics, Austin and Stuart (2015) contends that the KS statistic is a natural measure for assessing balance, while others (Ali et al., 2014) have shown that the KS statistic performs uniformly worse. Following Stuart et al. (2013), we will present pictorial comparisons of the GBM and CBPS, using both standardized bias, and KS statistics. As stated earlier, a significant novelty of the CBPS method is avoiding the iterative process between model fitting and balance checking, and presenting simultaneous implementation that maximizes covariate balance and prediction of subgroup assignment. However, this is not the case with GBM.

### 3.3 Stage II: Frailty Cox Model with Cure Rate

We first describe the causal model for the potential outcomes and then identification based on the PS weighted observed data likelihood. The frailty model is an extension of the Cox proportional hazards (PH) model (Lin and Wei, 1989) to account for unobserved heterogeneity, introduced via clustering among study subjects (within a transplant center), under the assumption that subjects within a specific cluster experience similar risks, and different from subjects in other cluster. Let $U_{i}$ be the (random) frailty effect corresponding to the ith transplant center, with the density $f_{\theta }(\cdot )$. We consider the Cox frailty model for the potential outcomes $X_{\text{ij}}(v)$, for v∈{D±/R±}. Considering $z={\left(z_{1},\ldots ,z_{V-1}\right)}^{\prime }$ the V-1 dummy indicators of treatment levels, the shared frailty $\text{exp}\left(u_{i}\right)$ is added as a linear term to the linear combination of the V-1 dummy indicators of group/treatment effects, leading to the frailty PH model for $X_{\text{ij}}(v)$:

$$h_{\text{ij}}(x|z)=h_{0}(x)\;\text{exp}\left(\beta^{T}z+u_{i}\right)$$

where, $h_{0}\left(x_{\text{ij}}\right)$ is the baseline hazard (see next subsection for the choices), and β is the vector of regression coefficients that depict the casual effects of treatments.

To motivate the observed data likelihood function, we consider the case when treatment is randomly assigned and we will then consider the PS weighting to address for selection bias due to non-random treatment assignment. Let $Z_{\text{ij}}={\left(Z_{\text{ij},1},\ldots ,Z_{\text{ij},V-1}\right)}^{\prime }$ be the V-1 dummy indicators of treatment levels. Our observed data is $𝒟=\left\{\left(X_{\text{ij}},\delta_{\text{ij}},W_{\text{ij}},Z_{\text{ij}}\right),:i=1,\ldots ,I,j=1,\ldots ,n_{i}\right\}$. When the treatment is randomly assigned, the likelihood for the ith center is given by:

(2) $$L_{i}=\int_{-\infty }^{\infty }\prod_{j=1}^{n_{i}}{\left[f\left(x_{\text{ij}}\right)\right]}^{\delta_{\text{ij}}}S{\left(x_{\text{ij}}|\cdot \right)}^{1-\delta_{\text{ij}}}f_{\theta }\left(u_{i}\right)du_{i}\;=\int_{-\infty }^{\infty }\prod_{j=1}^{n_{i}}{\left[h_{\text{ij}}\left(x_{\text{ij}}|Z_{\text{ij}}\right)\right]}^{\delta_{\text{ij}}}S\left(x_{\text{ij}}|\cdot \right)f_{\theta }\left(u_{i}\right)du_{i}$$

where, $S\left(x_{\text{ij}}|\cdot \right)=\text{exp}\left[-\int_{0}^{x_{\text{ij}}}h_{\text{ij}}\left(t|Z_{\text{ij}}\right)dt\right]$ is the survival probability at time $x_{\text{ij}}$ for those who have not died, and $\delta_{\text{ij}}$ is the censoring indicator.

When high-censoring is observed, a typical modeling assumption is the existence of a cure rate (Mirzaee et al., 2014) under the premise that a proportion of subjects would never experience the event of interest (here, recipient’s death). A popular model is the 2-component mixture cure model (Sy and Taylor, 2000), which separates the censored population into the cured and uncured group. The corresponding survival function (at time t) is given by:

$$S_{\text{pop}}(t|\cdot )=p_{\text{ij}}+\left(1-p_{\text{ij}}\right)S(t|\cdot )$$

where, $p_{\text{ij}}$ is the probability of a recipient being cured, ($1-p_{\text{ij}}$) be the proportion of uncured recipients, and S(t∣⋅) the survival probability at time t for the uncured recipients. The cure proportion, given covariates, can be estimated through the logistic link function

$$p_{\text{ij}}=p_{\text{ij}}\left(Z_{\text{ij}}\right)=\frac{1}{1+\text{exp}\left(-\alpha^{T}Z_{\text{ij}}-\lambda u_{i}\right)}$$

where, α in the parameter vector corresponding to treatment covariate $Z_{\text{ij}}$ in the logistic model, and $u_{i}$ the shared frailty between the logistic and frailty models, whose association is explained by the scalar λ. The observed data likelihood for our mixture cure frailty setup (corresponding to center i) now takes the form:

(3) $$L_{i}=\int_{-\infty }^{\infty }\prod_{j=1}^{n_{i}}{\left[\left(1-p_{\text{ij}}\right)h_{\text{ij}}\left(x_{\text{ij}}|Z_{\text{ij}}\right)S\left(x_{\text{ij}}|\cdot \right)\right]}^{\delta_{\text{ij}}}{\left[p_{\text{ij}}+\left(1-p_{\text{ij}}\right)S\left(x_{\text{ij}}|\cdot \right)\right]}^{1-\delta_{\text{ij}}}f_{\theta }\left(u_{i}\right)du_{i}$$

with $S\left(x_{\text{ij}}|\cdot \right)$ the survival probability at time $x_{\text{ij}}$ for uncured population. Note, the likelihood in (3) involves an integral (with respect to the random effects $u_{i}$), which can be handled using numerical integration techniques, such as the Gaussian quadrature.

#### Modeling Baseline Hazard:

The baseline hazard $h_{0}(.)$ can be left completely unspecified (nonparametric route), or modeled via the popular Weibull density (parametric route). The former involves non-parametric terms in the integrand which may impair the Gaussian quadrature routine (Liu and Huang, 2008), while the latter, being a restrictive (parametric) choice, compromises on the flexibility. Here, we choose the piecewise-constant form (He et al., 2013) for the baseline hazards as a pragmatic alternative, balancing flexibility and computational scalability. Following literature (Lawless and Zhan, 1998; Feng et al., 2005), we divide the follow-up time to survival into 10 intervals by every 10th quantile (denoted as $Q_{1},Q_{2},\ldots ,Q_{10}$, with $Q_{0}=0$, or the smallest event time) among the observed survival time. The piecewise-constant baseline hazard ${\tilde{h}}_{0}(t)$ now takes the form:

$${\tilde{h}}_{0}(t)=\sum_{k=1}^{10}h_{0k}I\left(Q_{k-1}<t⩽Q_{k}\right)$$

where, ${\tilde{h}}_{0}(t)=h_{0k}$ in the interval $Q_{k-1}<t⩽Q_{k}$, and I(.) is the indicator function. The corresponding cumulative baseline hazard is given as:

$${\tilde{H}}_{0}(t)=\sum_{k=1}^{10}h_{0k}\;\text{max}\;\left(0,\text{min}\left(Q_{k}-Q_{k-1},t-Q_{k-1}\right)\right)$$

#### Propensity Score Weighted Observed Likelihood:

Consider the full parameter vector $\Theta =\left(\beta ,\alpha ,\lambda ,\theta^{2}\right)$. Factoring in the inverse propensity estimate $\pi_{\text{ij}}^{\left(v\right)}$ from Stage I, the likelihood in (3) for the ith center, assuming time-independent covariates, takes the form:

(4) $$L_{i}\approx \int_{-\infty }^{\infty }\prod_{j=1}^{n_{i}}\text{exp}\left(\text{log}\left(\pi_{\text{ij}}^{\left(v\right)}\right)+\delta_{\text{ij}}\left[\text{log}\left(1-p_{\text{ij}}\right)+\text{log}\;{\tilde{h}}_{0}\left(x_{\text{ij}}\right)+\text{log}\;S\left(x_{\text{ij}}|\cdot \right)\right]\right.\left.+\left(1-\delta_{\text{ij}}\right)\left[\text{log}\left(p_{\text{ij}}+\left(1-p_{\text{ij}}\right)S\left(x_{\text{ij}}|\cdot \right)\right)\right]\right)f_{\theta }\left(u_{i}\right)du_{i}\approx \int_{-\infty }^{\infty }\prod_{j=1}^{n_{i}}\text{exp}\left(\tilde{l_{\text{ij}}}\right)f_{\theta }\left(u_{i}\right)du_{i}$$

where, $\tilde{l_{\text{ij}}}=\text{log}\left(\pi_{\text{ij}}^{\left(v\right)}\right)+\delta_{\text{ij}}\left[\text{log}\left(1-p_{\text{ij}}\right)+\text{log}\;{\tilde{h}}_{0}\left(x_{\text{ij}}\right)+\text{log}\;S\left(x_{\text{ij}}|\cdot \right)\right]+\left(1-\delta_{\text{ij}}\right)\left[\text{log}\left(p_{\text{ij}}+\left(1-p_{\text{ij}}\right)S\left(x_{\text{ij}}|\cdot \right)\right)\right]$

## 4 Estimation

### 4.1 Stage I: Estimating Propensity Scores

#### GBM:

For GBM-based estimation, we follow the suggestions as outlined in McCaffrey et al. (2004) for handling multiple exposure groups via creation of dummy indicators for the multiple exposure groups, and fitting separate GBMs to the dummy indicators to obtain the estimated PS for the given treatments in question. For a given exposure group, estimation of the corresponding PS would only require knowing the probability that each study subject assigned to this group indeed received the assignment (rather than any other exposures). This leads to a binary exposure situation, where the GBM fits efficiently balances between pretreatment characteristics, albeit with proper tuning, balance measures and stopping criterion to prevent over-fitting. Estimated propensity model and values using mean ES and max KS (as stopping rules) were both considered for propensity estimation. The GBM implementation (refer to codes in Appendix C.1) are available via the R twang package, which can also be called through a SAS macro (Ridgeway et al., 2020), available at https://www.rand.org/content/dam/rand/www/external/statistics/twang/twang_mac_v3.1.2.sas.

#### CBPS:

Following Imai and Ratkovic (2014), the GMM was used to estimate CBPS. Further methodological details on the related method of moments conditions and its estimation can be found in Imai and Ratkovic (2014). Implementation of CBPS (refer to code in Appendix C.2) are made available via the R CBPS package (Imai and Ratkovic, 2014).

### 4.2 Stage II: Estimation in the Cox Mixture Cure Frailty Setup

#### Gaussian Quadrature:

Within our frailty specification, we utilize an adaptive Gaussian quadrature (Pinheiro and Bates, 1995) to conveniently tackle the integration of the marginal maximum likelihood over random effects. Assuming the random frailty (center) effect $u_{i}$ with density $f_{\theta }\left(u_{i}\right)=f\left(u_{i}|\theta \right)$ following normal distribution $\text{N}\left(0,\theta^{2}\right)$, the likelihood in (4) can now be approximated by a weighted average of the integrand assessed at the Q=10 predetermined points $d_{q}(q=1,\ldots ,Q)$ over the random effects $u_{i}$. The likelihood expression $L_{i}$ now becomes:

$$L_{i}\approx \sum_{q=1}^{Q}\prod_{j=1}^{n_{i}}\text{exp}\left(\overset{ˆ}{l_{\text{ij}}}\right)f_{\theta }\left(d_{q}\right)w_{q}$$

where, $d_{q}=\sqrt{2}a_{q},w_{q}=\sqrt{2}\eta_{q}e^{a_{q}^{2}}$, with the standard Gauss-Hermite weights $\eta_{q}$ and abscissas $a_{q}$ obtained from available algorithms (Golub and Welsch, 1969), and $\overset{ˆ}{l_{\text{ij}}}$ obtained by replacing the random effect $u_{i}$ in the expression of $\tilde{l_{\text{ij}}}$ by the quadrature points $d_{q}$. We employ SAS Proc NLMIXED to accomplish the optimization steps through a dual quasi-Newton optimization (Nocedal and Wright, 2006). The optimization is automated within the NLMIXED procedure, which also allows user-desired specification of the log-likelihood function.

## 5 Application: UNOS Data

Of the 189,271 transplants conducted within 299 unique centers in the UNOS database, 176,962 (93.5%) belong to D−/R− pairing group, 7,735 (4.1%) to D−/R+, 746 (0.4%) to D+/R−, and the rest 3,828 (2.0%) to D+/R+. 36 subjects had invalid recipient survival time, censoring or strata values, leaving 189,235 kidney-related transplantation for our analysis. When conditioned on complete observations, there were 134,608 transplants (93.8%) for D−/R−, 5,408 (3.8%) for D−/R+, 416 (0.3%) for D+/R−, and 3000 (2.1%) for D+/R+. Median survivals (days) for the four exposure groups are as follows: 4345 (D−/R−), 3436 (D−/R+), 2420 (D+/R−), and 2923 (D+/R+). The well-separated censored survival curves for the 4 HCV groupings showed no indication of violation to the proportional hazards (PH) assumption. Furthermore, log-rank tests reveal that the 10-year recipient survivals between the 4 pairings were significantly different (p < 0.0001), with D+/R− having the lowest survival followed by the D+/R+ pairings.

**Figure 2:**
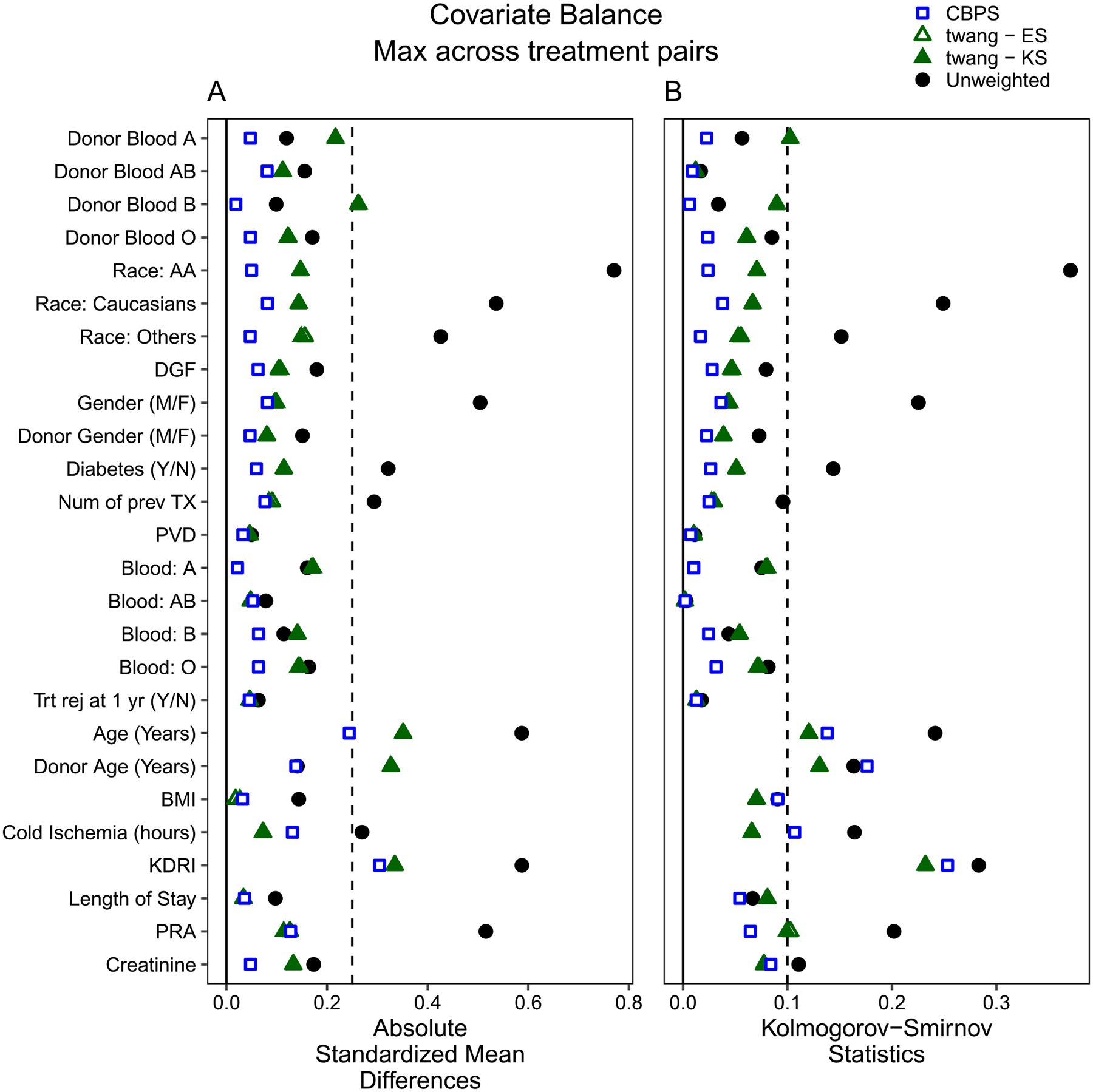
Love plots comparing propensity score balances between the unweighted, GBM (with effect size, ES stopping rule), GBM (with Kolmogorov-Smirnov, KS stopping rule), and the CBPS methods, using ASMD and KS statistics. For each covariate, the maximum of the ASMD and KS statistics corresponding to the 6 treatment/exposure pairs were considered. The GBM fits were implemented via twang. Other abbreviations include AA = African Americans; DGF = delayed graft function; PVD = peripheral vascular disease; BMI = body mass index; KDRI = kidney donor risk index; PRA = panel reactive antibodies.

After the PS estimation in Stage I modeling (using GBM and CBPS), we proceed to conduct the common support and balance assessments. With regards to assessing common support, the box-plots (see, Figure A1 in Supplementary Material) reveal distinct non-overlapping propensity distributions for the different HCV-DR groups. The problem is further confirmed (wrt. balance assessments), when we look closer at the balance table checks (Table B1 in Supplementary Material) where covariates are compared pairwise between the treatment/exposure groups for significant differences, separately for the unweighted and weighted cases. The application of the IPTW from the GBM showed imbalanced continuous covariates among the exposure groups. For the weighted cases, while the mean estimates of the continuous variables/covariates corresponding to the 4 subgroups are not that far apart, the minimum p-values for the pairwise comparisons corresponding to KDRI, donor age, and recipient age were significant, implying imbalance. Further balance assessments were considered using Love plots. Considering the ASMD statistic (see Figure 2, left panel), the plots corresponding to GBM reveal that regardless of the stopping rules (ES, or KS), on the overall, utilizing weights assisted in achieving balance for most covariates, compared to the unweighted versions. Despite these improvements, however, a few covariates such as donor age, recipient age, KDRI, and donor blood type (Type B) did not fall below the recommended ASMD threshold. On the other hand, the *CBPS* plots displayed improved balancing, where only the covariate KDRI missed the threshold. Similar conclusions were derived from the plots using the KS statistic (Figure 2, right panel), where, both the GBM and CBPS achieved improved balance (compared to the unweighted case), albeit some covariates, such as donor age, recipient age, and KDRI (which were also deemed unbalanced using AMSD). Henceforth, in our Stage-II survival modeling, we utilize inversely weighted PS estimated from the CBPS method.

During Stage-II modeling, we proceed via fitting 4 models (varying with/without the frailty, and with/without cure components) separately to the recipient survival endpoints. Looking at the model fit comparisons (see Table B2 in Supplementary Material), we observe that the frailty model with cure has the smallest AIC (2,083,251) and BIC (2,083,396), followed by the frailty model without cure (AIC: 2,085,239; BIC: 2,085,300). While literature (Burnham and Anderson, 2004) suggests a difference of 10 (or more) units to be indicative of a better fit (lower AIC/BIC is better), the differences observed in our case (between the two lowest AIC/BIC values) is approximately > 2000, implying that the model with frailty and cure assumptions produces the best fit. The models without frailty assumptions produces worse fits on all accounts.

From the fit of the unadjusted model (i.e. non-cure and non-frailty) to the recipient survival outcome (see Table B3 in Supplementary Material), we observe that the less conventional pairings D−/R+ (hazard ratios (HR): 1.59; 95% confidence interval (CI): 1.54, 1.63), D+/R− (HR: 1.77; 95% CI: 1.72, 1.82), and D+/R+ (HR: 1.42; 95% CI: 1.38, 1.46) have higher risk of death, compared to D−/R−. These results were similar for all four models. Looking at the cure model with frailty, the odds of being cured for D−/R+ (OR: 0.89; 95% CI: 0.65, 1.23), D+/R− (OR: 0.04; 95% CI: 0.01, 0.08) and D+/R+ (OR: 0.67; 95% CI: 0.55, 0.91) groups were all lower than D−/R− group. Again, we observe the same agreement in the odds ratio results with the cured and non-frailty model, where the odds ratios were all lower than 1 compared to conventional pairing. Finally, the estimate of λ is negative and significant (−4.26, 95% CI: −4.34, −4.17), which makes intuitive sense, given that a higher cure probability would imply lower survival hazards.

### 5.1 Sensitivity Analysis

We now conduct a sensitivity analysis to assess the robustness of our conclusions in presence of unmeasured confounders (Rosenbaum, 2010), focusing on the best-fitting model (frailty with cure). Existing methodologies for evaluating sensitivity can be divided into the semi-/non-parametric, and parametric approaches; for a discussion on the merits and inferiority of these approaches, see Carnegie et al. (2016). For our case with survival analytic endpoints which involves semi-parametric models, we follow the simulated unobserved confounder approach of Huang et al. (2020). Specifically, we introduce $M=M_{\text{ij}}$ as the vector of unmeasured confounders varying with center and subject with the corresponding coefficient/parameter ζ, independent of the vector of observed covariates $W_{\text{ij}}$ into the frailty Cox model. Furthermore, given $W_{\text{ij}}$ and $M_{\text{ij}}$, the group (dummy) indicator $Z_{\text{ij}}$ follows a generalized linear model, say a multinomial probit, with the coefficients $\gamma_{z}$ and $\zeta_{z}$ respectively for W and M. Note, ζ and $\zeta_{z}$ are the sensitivity parameters quantifying the relationships between the unobserved confounder **M** and the groups, and the confounder and right-censored response, respectively. Furthermore, we assume $M_{\text{ij}}\sim \text{Bernoulli}(0.5)$. Given the observed data, we thus simulate **M**. With the simulated **M**, we proceed with fitting the frailty Cox model with cure to the UNOS dataset for values of ζ∈{-2,-1,0,1,2} and $\zeta_{z}\in \{0,0.5,1,1.5,2\}$. This was easily implemented via our NLMIXED routine with Q=10 quadratures, with parameter estimates obtained from prior runs using observed covariates as our starting values.

It should be noted that Huang et al. (2020) provides the sensitivity assessment framework utilizing the EM, or the stochastic EM approach. However, attuned to Section 4, we resort to Gaussian quadrature based full maximization here. Results from our sensitivity analysis in terms of estimated HR and their standard errors are presented in Table B4 (Supplementary Material). When $\zeta_{z}=0$ and ζ shifted from 0 to ±2, departures were observed in the estimates corresponding to the D−/R+ group compared to the other groups. When $\zeta_{z}=2$, noticeable departures were observed for ζ=±2 (higher values of ζ), but estimates were close to the real data fit once ζ=0. However, these departures do not warrant differences in the overall conclusions of the HCVDR effects.

## 6 Conclusions

On the overall, models with the frailty term exhibit better fit than non-frailty models. Without losing the same inference than the usual cox PH model (non-frailty and non-cured model), the cure rate model with clustering feature provided the best fit (compared to the other models) for the recipient survivals, along with a narrower CI. The hazard ratios and odds ratios convey the same message for HCV groupings, i.e., the transplant recipients, in regards to D−/R+, D+/R− & D+/R+ have lower odds of being cured and higher risk of death (whether due to kidney failure) than compared to transplants with D−/R− pairings when looking at recipient outcomes. The inverse relationship between the OR and HR made clinical sense as those who are more likely to be cured have lower risk of death.

The low volume of transplants for the D+/R+ or D+/R− groups imply lower supply of these donor organs. Findings from previous studies (Gupta et al., 2017; Bucci et al., 2002) convey that the D+/R− group continues to have an inferior recipient survival, compared to their matched counterpart, the D−/R− group. Since R− recipients are receiving healthy transplants from D− (in the D−/R− group), they are healthy from the start. Conversely, recipients receiving (high risk) hepatitis C positive organs (D+/R−, and D+/R+ groups) are expected to exhibit the lowest (raw) survival probabilities. Biologically, those who receive Hepatitis C are at a higher risk (Scott et al., 2010) of infection transmission and post-transplant diabetes and glomerulonephritis (Cacoub et al., 2016). Simply put, the D+/R− pairing, that constituted healthy individuals with unhealthy organs, had the lowest survival and highest mortality risk. Thus, the low amount of transplants for D+/R− seems warranted. A similar result showed that the D+/R+ cohort was significantly associated with increased mortality, compared to the D−/R− reference (Singh et al., 2012). To date, the only therapy available for HCV was through interferon which was shown to have increased risk of kidney allograft rejection (Wei et al., 2014; Carbognin et al., 2006). It was also revealed that donor-positive HCV-status was associated with increased mortality regardless of recipients’ HCV-status, in their adjusted and unadjusted analysis using multivariate Cox proportional hazards model, with subjects censored at 10 years. It should be noted that sub analyses from Gupta et al. (2017) found that the 5-year recipient survival of the D+/R− group was superior to waitlisted controls.

In order to utilize the powerful Gaussian quadrature technique, one must consider some facets for a successful estimation. First, proper starting values of the parameters are necessary for convergence of the Gaussian quadrature routine. Plausible starting values can be obtained by fitting a simple Cox models (ignoring the random effect) for the survival model, and a logistic regression for the cure rate model. We assessed our fit using various starting values. The resulting estimates (after converge of the algorithm) were very close, implying some degree of robustness to the starting values. Next, one needs to decide on the number of quadrature points. Following the recommendation of Liu and Huang (2008), we used 10 intervals. Table B5 (see Supplementary Materials) presents estimates from the data refit using 5 quadrature points. Although some changes in parameter estimates were noted (as expected), the overall significance and interpretation remained unchanged. Based on our experience, too few points may result in non-convergence of the quadrature technique, and too many points can unnecessarily complicate the NLMIXED framework.

A few limitations exist in our proposed analysis. First, the ‘black-box’ nature of the GBM (and the corresponding twang implementation) only provides the PS (or, probability of being in an exposure group for each observation), and prevents evaluation of specific covariate effects during the Stage-I PS modeling. Next, we observe that balance was not achieved for some covariates (under both GBM, and/or CBPS), such as the KDRI, although some of which were revealed to be key factors (Gill et al., 2008; Gupta et al., 2017; Ojo et al., 2001; Gill et al., 2009; Kamal et al., 2020) determining survival. Hence, instead of outright deletion, they were adjusted in the Stage-I modeling. Furthermore, while we controlled for a few confounders, we cannot rule out the possibility of other unmeasured confounders. Also, the significant imbalance (in frequency) among the exposure groups was not directly addressed in our current modeling. Finally, to avoid issues with non-convergence, starting values should be carefully chosen during the implementation of the NLMIXED procedure in Stage-II. All these are part of future work, and will be pursued elsewhere. Nevertheless, our proposed methodology is applicable to other biomedical observational data settings, with the goal of quantifying the associations between survival outcomes and treatment/exposure groups.

Our current framework rests upon the unmeasured confounding assumption, which is not verifiable from the observed data. We used sensitivity analysis (Subsection 5.1) to assess the extent to which the inference derived is robust to violations of the assumption. We may also consider the PS approach of Yang (2018) that allows unmeasured cluster-level confounders. PS weighting can be unstable if some propensity score estimates are close to zero; in this case, propensity score matching for multi-level treatments (Yang et al., 2016) can be considered. We plan to consider these topics for future research. Also, in the current study, the treatment/group indicator is assigned at the baseline, and does not vary in the follow up, restricting confounding only at the baseline. In this case, adjusting for baseline confounders is sufficient to remove confounding bias. However, in longitudinal observational studies with time-varying treatment, one needs to adjust for time-varying confounders; see, e.g. Yang et al. (2018, 2020) for causal analyses of survival outcomes in these settings.

## Supplementary Material

Supplementary Materials

Figures and Tables pertaining to the motivating UNOS data analysis, as well as computing code (in R and SAS) and a representative UNOS dataset consisting of a random sample with 19000 observations are available as accompanying supplementary materials associated with this paper.
