# Supplementary material for: Propensity Score Modeling in Electronic Health Records with Time-to-Event Endpoints: Application to Kidney Transplantation
Source: J Data Sci. Author manuscript; Available in PMC 2026 Aug 6. (PMC13441248; doi:10.6339/22-jds1046)
Supplement: Supplementary file 1 [file NIHMS2197459-supplement-Supplementary_Materials.zip › JDS1046_Supplementary.pdf]

Supplementary Materials for "Propensity score modeling in electronic health records with time-to-event endpoints: Application to kidney transplantation"

Jonathan W. Yu[1], Dipankar Bandyopadhyay[1,*], Shu Yang[2], Le Kang[1] and Gaurav Gupta[3]

[1]Department of Biostatistics, Virginia Commonwealth University, Richmond, VA
[2]Department of Statistics, North Carolina State University, Raleigh, NC
[3]Division of Nephrology, Virginia Commonwealth University, Richmond, VA

[*]*Address of Correspondence*: One Capitol Square, 7th Floor, 830 East Main Street, PO Box 980032, Richmond, VA, 23298-0032, USA. Tel: +1 (804) 827-2058; E-mail: bandyopd@gmail.com

A. Figures

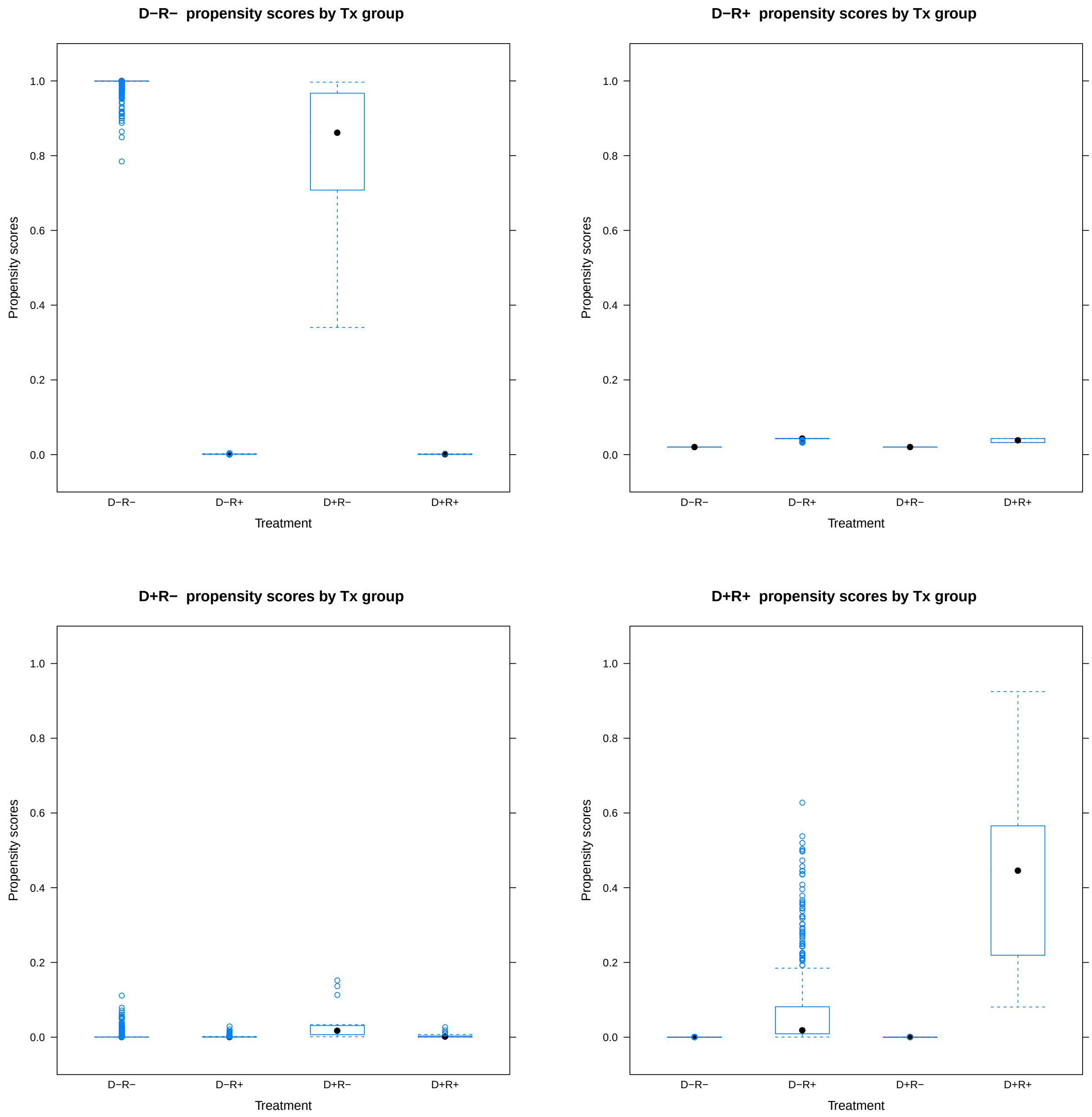

Figure A1: Propensity score diagnostic checks for proper support output from the GBM using the `R` `twang` package.

B. Tables

	Unweighted					Weighted				
	D+/R−	D+/R+	D−/R−	D−/R+	p^*	D+/R−	D+/R+	D−/R−	D−/R+	p^*
Potential continuous confounders										
Age	57.03	55.25	50.08	51.88	<0.001	52.38	54.42	50.26	50.62	<0.001
Donor age	39.11	39.46	37.46	37.87	<0.001	35.38	40.09	37.52	37.82	0.030
Length of stay	8.18	7.45	7.77	8.53	<0.001	7.47	7.84	7.79	7.78	0.548
BMI	27.33	27.16	27.64	26.89	<0.001	27.57	27.51	27.60	27.59	0.742
Creatinine	3.32	3.77	3.51	3.81	<0.001	3.26	3.64	3.52	3.44	0.070
Kidney donor risk index	1.24	1.18	0.95	0.96	<0.001	1.29	1.37	1.23	1.24	<0.001
Previous number of treatment	0.088	0.094	0.126	0.183	<0.001	0.10	0.12	0.13	0.13	0.283
Panel reactive antibodies	10.73	9.31	19.41	24.46	<0.001	15.92	16.05	19.39	19.75	0.090
Hours in cold ischemia	20.29	19.48	17.88	18.20	<0.001	17.50	18.19	17.93	17.98	0.460

Table B1: Balance table between the continuous variables from the GBM via the `twang` package. The minimum of p-values are provided across all pairwise comparisons between covariates among the HCVDR groups. Indication of significant p-values imply imbalance.

* Smallest pairwise difference test.

Propensity score method	Models	AIC	BIC	-2log-lik
	Recipient survival			
	Non-clustered Model w/o cure rate	2,101,365	2,101,425	2,101,353
IPW	Non-clustered Model w/ cure rate	2,101,359	2,101,419	2,101,347
	Clustered Model w/o cure	2,085,239	2,085,300	2,085,227
	Clustered Model w/ cure	2,083,251	2,083,396	2,083,323

Table B2: Model fit comparison between models with/without frailty, and with/without cure rate assumptions.

Logistic	Frailty				Non-frailty			
	Cured		Non-cured		Cured		Non-cured	
	OR (95% CI)	P-val	OR (95% CI)	P-val	OR (95% CI)	P-val	OR (95% CI)	P-val
HCV Groupings [a]								
$D-/R+$	0.89 (0.65, 1.23)	0.32	–	–	0.4 $(0.0, \infty)$	0.99	–	–
$D+/R-$	0.04 (0.01, 0.08)	$< 0.0001^*$	–	–	0.07 $(0.0, \infty)$	0.99	–	–
$D+/R+$	0.67 (0.55, 0.91)	0.03	–	–	0.27 $(0.0, \infty)$	0.98	–	–
Survival	HR (95% CI)	P-val	HR (95% CI)	P-val	HR (95% CI)	P-val	HR (95% CI)	P-val
HCV Groupings [a]								
$D-/R+$	1.61 (1.57, 1.66)	$< 0.0001^*$	1.64 (1.56, 1.69)	$< 0.0001^*$	1.59 (1.54, 1.63)	$< 0.0001^*$	1.68 (1.63, 1.76)	$< 0.0001^*$
$D+/R-$	1.75 (1.71, 1.79)	$< 0.0001^*$	1.83 (1.79, 1.87)	$< 0.0001^*$	1.77 (1.72, 1.82)	$< 0.0001^*$	1.88 (1.76, 2.01)	$< 0.0001^*$
$D+/R+$	1.46 (1.40, 1.52)	$< 0.0001^*$	1.38 (1.34, 1.42)	$< 0.0001^*$	1.42 (1.38, 1.46)	$< 0.0001^*$	1.43 (1.35, 1.51)	$< 0.0001^*$
	$\hat{\beta}$ (SD)	P-val	$\hat{\beta}$ (SD)	P-val	$\hat{\beta}$ (SD)	P-val	$\hat{\beta}$ (SD)	P-val
λ	-4.26 $(-4.34, -4.17)$	$< 0.0001^*$	–	–	–	–	–	–
θ^2	0.56 (0.52, 0.61)	$< 0.0001^*$	0.28 (0.26, 0.30)	$< 0.0001^*$	–	–	–	–

[a] D–/R– as base group; * p-val < 0.0001

Table B3: UNOS data (model fitting): Parameter estimates, 95% confidence intervals, and p-values, corresponding to fitting the 4 models varying with frailty/non-frailty and cure/non-cure assumptions to the recipient survival outcome using 10 quadrature points.

ζ	Method	Group	$\zeta^z = 0$	$\zeta^z = 0.5$	$\zeta^z = 1$	$\zeta^z = 1.5$	$\zeta^z = 2$
		D−/R+	1.70 (0.054)	1.98 (0.063)	2.06 (0.065)	2.86 (0.069)	3.42 (0.075)
2	IPW	D+/R−	1.74 (0.076)	2.11 (0.078)	2.84 (0.089)	3.79 (0.100)	4.68 (0.106)
		D+/R+	1.48 (0.072)	1.66 (0.078)	1.74 (0.080)	2.23 (0.092)	2.97 (0.104)
		D−/R+	1.69 (0.047)	1.74 (0.050)	1.88 (0.057)	2.37 (0.065)	2.81 (0.072)
1	IPW	D+/R−	1.75 (0.043)	1.82 (0.049)	2.37 (0.055)	2.98 (0.060)	3.92 (0.069)
		D+/R+	1.46 (0.066)	1.53 (0.069)	1.60 (0.071)	2.13 (0.075)	2.71 (0.087)
		D−/R+	1.69 (0.044)	1.70 (0.046)	1.70 (0.063)	1.70 (0.063)	1.70 (0.063)
0	IPW	D+/R−	1.75 (0.040)	1.76 (0.048)	1.78 (0.050)	1.78 (0.051)	1.79 (0.053)
		D+/R+	1.46 (0.061)	1.46 (0.061)	1.45 (0.061)	1.45 (0.061)	1.45 (0.061)
		D−/R+	1.69 (0.047)	1.67 (0.050)	1.70 (0.058)	1.70 (0.064)	1.70 (0.070)
-1	IPW	D+/R−	1.75 (0.046)	1.47 (0.048)	1.89 (0.053)	1.89 (0.059)	1.89 (0.065)
		D+/R+	1.46 (0.064)	1.23 (0.067)	1.23 (0.069)	1.23 (0.074)	1.02 (0.085)
		D−/R+	1.70 (0.052)	1.61 (0.063)	1.42 (0.064)	1.18 (0.067)	1.02 (0.071)
-2	IPW	D+/R−	1.74 (0.069)	1.70 (0.079)	1.61 (0.088)	1.32 (0.101)	1.21 (0.107)
		D+/R+	1.48 (0.068)	1.42 (0.079)	1.36 (0.081)	1.05 (0.094)	0.99 (0.100)

Table B4: Sensitivity assessments to unmeasured confounder effect in the UNOS dataset for values of $\zeta \in \{-2, -1, 0, 1, 2\}$ and $\zeta_z \in \{0, 0.5, 1, 1.5, 2\}$ under the cure frailty model. Table entries are the estimated Hazard Ratios (Standard Errors) for the three groups, considering D−/R− as the baseline group.

	Frailty				Non-frailty			
Logistic	Cured		Non-cured		Cured		Non-cured	
	OR (95% CI)	P-val	OR (95% CI)	P-val	OR (95% CI)	P-val	OR (95% CI)	P-val
HCV Groupings [a]								
$D-/R+$	0.92 $(0.61, 1.28)$	0.23	–	–	0.3 $(0.0, \infty)$	1.00	–	–
$D+/R-$	0.09 $(0.02, 0.16)$	$< 0.0001^*$	–	–	0.11 $(0.0, \infty)$	1.00	–	–
$D+/R+$	0.48 $(0.55, 0.91)$	0.05	–	–	0.18 $(0.0, \infty)$	1.00	–	–
Survival	HR (95% CI)	P-val	HR (95% CI)	P-val	HR (95% CI)	P-val	HR (95% CI)	P-val
HCV Groupings [a]								
$D-/R+$	1.68 $(1.59, 1.75)$	$< 0.0001^*$	1.68 $(1.63, 1.72)$	$< 0.0001^*$	1.44 $(1.26, 1.59)$	0.002^*	1.52 $(1.44, 1.61)$	$< 0.0001^*$
$D+/R-$	1.75 $(1.71, 1.79)$	$< 0.0001^*$	1.91 $(1.84, 1.99)$	$< 0.0001^*$	1.72 $(1.65, 1.80)$	0.001^*	1.86 $(1.73, 2.00)$	$< 0.0001^*$
$D+/R+$	1.46 $(1.40, 1.52)$	$< 0.0001^*$	1.33 $(1.31, 1.36)$	$< 0.0001^*$	1.42 $(1.38, 1.46)$	0.002^*	1.59 $(1.37, 1.75)$	$< 0.0001^*$
	$\hat{\beta}$ (SD)	P-val	$\hat{\beta}$ (SD)	P-val	$\hat{\beta}$ (SD)	P-val	$\hat{\beta}$ (SD)	P-val
λ	-8.77 $(-3.68, -13.44$	$< 0.0001^*$	–	–	–	–	–	–
θ	1.21 $(0.58, 1.87)$	$< 0.0001^*$	0.36 $(0.27, 0.59)$	$< 0.0001^*$	–	–	–	–

[a] D–/R– as base group; * p-val < 0.0001

Table B5: UNOS data (model fitting): Parameter estimates, 95% confidence intervals, and p-values, corresponding to fitting the 4 models varying with frailty/non-frailty and cure/non-cure assumptions to the recipient survival outcome using 5 quadrature points.

C. SAS/R codes

C1 Stage 1: GBM propensity estimation (SAS code)

```
/% SAS GBM macro: https://www.rand.org/statistics/twang/mnps-sas-tutorial.html %/
%mnps(treatvar = hcvdr,
      vars = age pra los bmi_calc serum_creat perip_vasc_new kdri TRTREJ1Y_KI ABO
       prev_tx_num hep_c dialysis_at_txp first_week_dial genderd_num gender_num
       cold_isch_ki eth_group_cat d_ABO d_age,
      class = prev_tx_num hep_c dialysis_at_txp first_week_dial genderd_num
       gender_num eth_group_cat d_ABO ABO,
      dataset = local.analysis,
      estimand = ATT,
      stopmethod = es.mean ks.max,
      ntrees = 5000,
      output_dataset=local.gbm_output_data,
      return_ps=TRUE,
      plotname=mnps_plot.pdf,
      Rcmd=C:\ ..\ R.exe,
      objpath=C:\ ..\ twang_mnps3);
```

C2 Stage 1: CBPS propensity estimation (R code)

```
library(CBPS)
data <- read.csv("final_data.csv")
full.covariates <- data[complete.cases(data[,c("AGE","GENDER","PRA","r_Blood",
"KDRI","PVD","COLD_ISCH_KI","prev_tx_num","eth_group_cat","genderd_num",
"don_Blood","AGE_DON","first_week_dial","LOS","has_diab","SERUM_CREAT",
"BMI_CALC","TRTREJ1YKI")]),]

CBPS.full <- CBPS(hcvdr~PRA+r_Blood+AGE+GENDER+KDRI+prev_tx_num+PVD+
                    eth_group_cat+genderd_num+don_Blood+AGE_DON+COLD_ISCH_KI+
                    first_week_dial+LOS+has_diab+SERUM_CREAT+BMI_CALC+
                    TRTREJ1YKI,
                    data=full.covariates )
```

C3 Stage 2: clustered events without cure-rate (SAS code)

```
ods output FitStatistics=fit3;
proc nlmixed data=kidney qpoints=170 noad;
parms beta1 = 0.98189 beta2 = 0.98189 beta3 = 0.98189
  alpha1 = 0.1 alpha2 = 0.1  alpha3 = 0.1 theta=4.7
  r01-r10 1e-4;
bounds r01 r02 r03 r04 r05 r06 r07 r08 r09 r10 theta  >= 0;
base_haz = r01*fail_r1 + r02*fail_r2 + r03*fail_r3 + r04*fail_r4 + r05*fail_r5
+ r06*fail_r6 + r07*fail_r7 + r08*fail_r8 + r09*fail_r9 + r10*fail_r10;
cum_base_haz = r01*dur_r1 + r02*dur_r2 + r03*dur_r3 + r04*dur_r4 + r05*dur_r5
+ r06*dur_r6 + r07*dur_r7 + r08*dur_r8 + r09*dur_r9 + r10*dur_r10;
* log-survival prob *;
mu = beta1*(hcvdr_num=1) + beta2*(hcvdr_num=2)+ beta3*(hcvdr_num=3) + nu;
ll = -cum_base_haz*exp(mu);
if pstatus=0 then loglik = ll;
if pstatus=1 then loglik = ll + log(base_haz) + mu;
model ptime~general(loglik*cbps_weight);
random nu~normal(0,theta) subject=ctr_code;
ods exclude iterhistory parameters;
run;
```

C4 Stage 2: clustered events with cure-rate (SAS code)

```
ods output FitStatistics=fit4;
proc nlmixed data=kidney qpoints=170 noad;
parms beta1  = 0.98189 beta2  = 0.98189 beta3  = 0.98189
  alpha1 = 0.1 alpha2 = 0.1 alpha3 = 0.1
  theta=4.7 lambda = 0.3
  r01-r10 1e-4;
bounds r01 r02 r03 r04 r05 r06 r07 r08 r09 r10 theta  >= 0;
base_haz = r01*fail_r1 + r02*fail_r2 + r03*fail_r3 + r04*fail_r4 + r05*fail_r5
+ r06*fail_r6 + r07*fail_r7 + r08*fail_r8 + r09*fail_r9 + r10*fail_r10;
cum_base_haz = r01*dur_r1 + r02*dur_r2 + r03*dur_r3 + r04*dur_r4 + r05*dur_r5
+ r06*dur_r6 + r07*dur_r7 + r08*dur_r8 + r09*dur_r9 + r10*dur_r10;
* cure *;
check = (alpha1 * (hcvdr_num=1)) +
(alpha2 * (hcvdr_num=2)) + (alpha3 * (hcvdr_num=3)) + lambda*nu;
expcheck = exp(-check);
    prob= 1/(1+expcheck);
* log-survival prob *;
mu = beta1*(hcvdr_num=1) + beta2*(hcvdr_num=2)+ beta3*(hcvdr_num=3)  + nu;
ll = -cum_base_haz*exp(mu);
if pstatus=0 then loglik = log(prob + (1-prob) * exp(ll));
if pstatus=1 then loglik = log(1-prob) + log(base_haz) + ll + mu;
model ptime~general(loglik*cbps_weight);
random nu~normal(0,theta) subject=ctr_code;
ods exclude iterhistory parameters;
run;
```
