# Supplementary material for: Propensity Score Modeling in Electronic Health Records with Time-to-Event Endpoints: Application to Kidney Transplantation
Source: J Data Sci. Author manuscript; Available in PMC 2026 Aug 6. (PMC13441248; doi:10.6339/22-jds1046)
Supplement: Supplementary file 1 [file NIHMS2197459-supplement-Supplementary_Materials.zip › JDS_Code.zip!/JDS_Code/SampleData_Creation.html]

JDS sample data creation from raw kidney data


# JDS sample data creation from raw kidney data

#### Yu, Jonathan W et. al

#### February 20, 2022

```
# Libraries
pacman::p_load(tidyverse,survival, ggplot2, survival, tidyverse, survminer, here)
```

# Read in raw UNOS kidney data

```
input.data <- here("raw_kidney_aim1.csv") 
data = read_csv(input.data) %>% 
  tibble()
```

```
## Warning: One or more parsing issues, see `problems()` for details
```

```
## Rows: 189271 Columns: 478
```

```
## -- Column specification --------------------------------------------------------
## Delimiter: ","
## chr (201): hcvdr, WL_ORG, USE_WHICH_PRA, DONATION, ON_DIALYSIS, ANTIBODY_TES...
## dbl (198): NUM_PREV_TX, CURRENT_PRA, PEAK_PRA, CREAT_CLEAR, GFR, MAX_KDPI_LO...
## lgl  (79): COD_WL, COD_OSTXT_WL, C_PEPTIDE, C_PEPTIDEDATE, CITIZEN_COUNTRY, ...
```

```
## 
## i Use `spec()` to retrieve the full column specification for this data.
## i Specify the column types or set `show_col_types = FALSE` to quiet this message.
```

# Cleaning

```
data = data %>% 
  mutate(
    eth_group_cat = as.factor(case_when(
      eth_group == 1 ~ "Caucasians",
      eth_group == 2 ~ "AfricanAmericans",
      TRUE ~ "Others"
      )),
    
    has_diab = as.factor(ifelse(DIAB %in% c(1,5,998), "NoDiabetes","Diabetes")),
    
    r_Blood = as.factor(case_when(
      ABO %in% c("A","A1","A2") ~ "A",
      ABO %in% c("A2B","A1B") ~ "AB",
      ABO %in% c("O") ~ "O",
      TRUE ~ "B"
      )),
    
    don_Blood = as.factor(case_when(
      ABO_DON %in% c("A","A1","A2") ~ "A",
      ABO_DON %in% c("A2B","A1B") ~ "AB",
      ABO_DON %in% c("O") ~ "O",
      TRUE ~ "B"
      )),
    
    PVD = as.factor(ifelse(perip_vasc_new == 1, "Yes","No")),
    
    hcvdr_num = as.factor(case_when(
      hcvdr == "D+R+" ~ 3,
      hcvdr=="D+R-" ~ 2,
      hcvdr=="D-R+" ~ 1,
      TRUE ~ 0
      ))
  )

full.covariates <- data %>% 
  select(REGION,CTR_CODE,TRR_ID_CODE,PTIME,PSTATUS,GTIME_KI,GSTATUS_KI,
         ptime_10yr,pstatus_10yr,gtime_10yr,gstatus_10yr,
         hcvdr,hcvdr_num,
         AGE,GENDER,PRA,r_Blood,KDRI,PVD,COLD_ISCH_KI,
         prev_tx_num,eth_group_cat,genderd_num,
         don_Blood,AGE_DON,first_week_dial,
         LOS,has_diab,SERUM_CREAT,BMI_CALC,TRTREJ1YKI) %>% 
  drop_na()
```

# Random sample 19,000 observations

```
JDS_data = full.covariates %>% sample_n(19000)
```

# Save data

```
JDS_data %>% 
  write_csv("JDS_Data.csv")
```
